# The two glycolytic markers GLUT1 and MCT1 correlate with tumor grade and survival in clear-cell renal cell carcinoma

**DOI:** 10.1371/journal.pone.0193477

**Published:** 2018-02-26

**Authors:** Damien Ambrosetti, Maeva Dufies, Bérengère Dadone, Matthieu Durand, Delphine Borchiellini, Jean Amiel, Jacques Pouyssegur, Nathalie Rioux-Leclercq, Gilles Pages, Fanny Burel-Vandenbos, Nathalie M. Mazure

**Affiliations:** 1 Nice University Hospital, Department of Pathology, Nice, France; 2 UCA, Université Côte d’Azur, Nice-Sophia-Antipolis, Institute for Research on Cancer and Aging of Nice, CNRS-UMR 7284-Inserm U1081, Nice, France; 3 Centre Scientifique de Monaco (CSM), Monaco, Monaco; 4 Nice University Hospital, Department of Urology, Nice, France; 5 Antoine Lacassagne Cancer Center, Department of Oncology, Nice, France; 6 Rennes University Hospital, Department of Pathology, Rennes, France; University of Nebraska Medical Center, UNITED STATES

## Abstract

**Background:**

Clear-cell renal cell carcinoma (ccRCC) is the most common type of kidney cancer. Although ccRCC is characterized by common recurrent genetic abnormalities, including inactivation of the von Hippel-Lindau (*vhl*) tumor suppressor gene resulting in stabilization of hypoxia-inducible factors (HIFs), the tumor aggressiveness and outcome of ccRCC is variable. New biomarkers are thus required to improve ccRCC diagnosis, prognosis and therapeutic options. This work aims to investigate the expression of HIF and proteins involved in metabolism and pH regulation. Their correlation to histoprognostic parameters and survival was analyzed.

**Methods:**

ccRCC of 45 patients were analyzed. HIF-1α, HIF-2α, HAF, GLUT1, MCT1, MCT4, CAIX and CAXII expression was assessed by immunohistochemistry in a semi-quantitative and qualitative manner. The GLUT1, MCT1, MCT4, CAIX and CAXII mRNA levels were analyzed in an independent cohort of 43 patients.

**Results:**

A significant correlation was observed between increased GLUT1, MCT1, CAXII protein expression and a high Fuhrman grade in ccRCC patients. Moreover, while HIF-1α, HIF-2α and HAF expression was heterogenous within tumors, we observed and confirmed that HIF-2α co-localized with HAF.

We confirmed, in an independent cohort, that GLUT1, MCT1 and CAXII mRNA levels correlated with the Fuhrman grade. Moreover, we demonstrated that the high mRNA level of both MCT1 and GLUT1 correlated with poor prognosis.

**Conclusions:**

This study demonstrates for the first time a link between the aggressiveness of high- Fuhrman grade ccRCC and metabolic reprogramming. It also confirms the role of HIF-2α and HAF in tumor invasiveness. Finally, these results demonstrate that MCT1 and GLUT1 are strong prognostic markers and promising therapeutic targets.

## Introduction

Renal carcinomas represent 3% of solid tumors and are the sixth leading cause of cancer death. The most common is clear-cell renal cell carcinoma (ccRCC), which is a unique model of solid tumors characterized by recurrent genetic abnormalities on the 3p25–26 locus resulting in inactivation of the von Hippel-Lindau (*vhl*) tumor suppressor gene. Despite the existence of this common mechanism of inactivation, these tumors are morphologically heterogeneous. The architecture can be solid, alveolar or acinar. The tumor cell cytoplasm is mostly clear, but a granular eosinophilic cytoplasm can be found and some cells are fusiform. There is also heterogeneity in their response to treatment. Highly costly anti-angiogenic targeted therapies are related to a high rate of morbidity, which has highlighted the need to establish new criteria for the definition of prescription and predictive factors of response. To date, the Fuhrman grade [1], defined in terms of the nuclear morphology of tumor cells, is the prognostic factor used routinely worldwide for grading renal cell carcinoma. It has been demonstrated to be the most powerful histoprognostic parameter able to predict cancer specific survival regardless of the pathological stage, although no link with a biological process has been established. However, the Fuhrman grade is criticized regarding its reproducibility and accuracy.

Inactivation of the *vhl* gene, which translates into a deficit in the VHL protein (pVHL), is the initial event in tumorigenesis of ccRCC [2]. pVHL functions as part of an E3 multiprotein ubiquitin ligase complex that targets the hypoxia-inducible factor-α (HIF-α) for proteosomal degradation. Thus, the absence of pVHL results in HIF stabilization, increased target expression irrespective of the oxygen concentration and gives a proliferative advantage to tumor cells. Stabilization of the HIF-α subunits is mainly due to specific post-translational modification. However, additional mechanisms have been identified. Koh *et al*. showed that the hypoxia-associated factor (HAF), an E3 ubiquitin ligase, binds to HIF-1α to promote its ubiquitination, regardless of the level of oxygen and pVHL [3]. Yet, HAF interaction with HIF-2α increases its transcriptional activity. These results suggest that HAF, overexpressed in various tumor types, is an essential element in the establishment of a tumor switch in which the tumor acquires a more aggressive phenotype due to transition of expression of HIF-1α to HIF-2α [4]. Moreover, HIF-1α and HIF-2α play non-redundant roles. HIF-1 appears to drive genes involved in metabolism, whereas HIF-2 drives the expression of genes encoding pro-survival factors [5]. These distinct roles have been mostly defined in VHL-deficient RCC cells in which HIF-2 has been shown to be necessary and sufficient to maintain tumor growth.

As ccRCC are glycolytic and lipogenic tumors [6], we focused attention on the metabolic HIF-target genes. The glucose transporter 1 (*glut1*), carbonic anhydrase 9 (*ca9*) and 12 (*ca12*) and monocarboxylate transporter 4 (*mct4*) are downstream targets of HIF involved in glycolysis and intracellular pH (pHi) homeostasis. GLUT1 is often expressed in aggressive and/or hypoxic tumors reflecting an exacerbated need for nutrients to support endless proliferation. On the other hand, the carbonic and lactic acid produced by the glycolytic pathway must be rapidly exported out of cancer cells to maintain viability and proliferation. CAs and MCTs (both lactate/H+ symporters MCT1 and the hypoxia-inducible MCT4) are associated with poor prognostic factors in many cancers.

To identify new tools to adjust and improve prognosis, diagnosis and treatment in ccRCC in parallel to the Fuhrman grade, we present a study characterizing changes in the expression of the two different isoforms of HIF and the HAF modulating protein, in addition to changes in the expression of proteins involved in metabolism related to the Fuhrman grade.

## Materials and methods

### Patients and tissue handling

Tissue samples from 73 patients with ccRCC that had undergone surgery in the urology department of the Nice University Hospital between May 2006 and March 2009 were selected (IHC cohort). As defined by the 2016 World Health Organization criteria, diagnosis was based upon pathology and cytogenetic analysis. To compare each group of Fuhrman grade, we selected 15 cases in all 3 groups (45 patients) corresponding to Fuhrman grades II, III and IV (Table 1). Initial management of surgical specimens was performed according to a standardized protocol. The surgical specimens were obtained immediately after nephrectomy. Fresh samples were collected for genetic examination. Tumor tissue was formol fixed within 1h and for 72h. All haematoxylin and eosin stained sections were reviewed by 2 uropathologists for confirmation of the original diagnosis and grade of each case. Blocks were considered representative of the tumor if they harbored the contingent of the highest grade and if they also included non-tumor kidney tissue used as endogenous immunohistochemistry (IHC) controls for some markers. For each block, the lowest and highest grades exhibited on the slide were noted.

**Table 1:** Summary of clinicopathological parameters of the IHC cohort.

Variables	Screening cohort (n = 45)	Fuhrman grade II (n = 15)	Fuhrman grade III (n = 15)	Fuhrman grade IV (n = 15)
**Mean age**	63.6	60.5	64.3	65.9
**Sex**				
**Male**	30 (67%)	10 (67)%	12 (80%)	8 (54%)
**Female**	15 (33%)	5 (33)%	3 (20)%	7 (46)%
**Pathological stage**				
**pT1a**	12 (27%)	9 (60%)	2 (13%)	1 (7%)
**pT1b**	8 (18%)	3 (20%)	4 (27%)	1 (7%)
**pT2b**	1 (2%)	0	1 (7%)	0
**pT3a**	22 (49%)	3 (20%)	7 (47%)	12 (80%)
**pT3b**	2 (4%)	0	1 (7%)	1 (7%)
**Mean diameter**	5.4	3.2	5.9	7.2

Informed consent was obtained from all individual participants included in the study. All patients gave written consent for the use of tumor samples for research. The study included only the major patients. All of the samples are the property of the tissue collection of the Pathology department, which are declared annually to the French Health Ministry. The procedures followed were approved by the institutional review board of the University Hospital of Nice. This study was conducted in accordance with the Declaration of Helsinki.

### Immunohistochemistry

IHC was performed on 2μm-thick sections. Immunolabeling and detection were performed using a Dako Autostainer AutoMate, as per the manufacturer’s recommendations. The antibodies used were against HIF-1α (generated in our laboratory [7]), HIF-2α (Novus, nb100-122), HAF (Abcam, AB95957), GLUT1 (Abcam, AB53654), MCT1 (generated in our laboratory [8]), MCT4 (Millipore, AB3316P), CAIX (generated in our laboratory [8]), CAXII (Sigma, HPA008773). The detection was performed using the Envision Flex Kit (Dako), with 3–3′ diaminobenzidine as a chromogen.

### IHC evaluation

Semi-quantitative analysis of the IHC was performed by two readers independently, with proofreading of cases for which results were discordant. A reading grid was established for each antibody. Expression of each protein was evaluated in the highest grade zone of the tumor represented on the slide. When different grades were present, the expression level of the lowest grade was also evaluated. For HIF-1α, HIF-2α and HAF, semi-quantitative analysis was done using the validated Allred score [9]. The quality, homogeneity and heterogeneity of the staining was analyzed and it was noted if the staining of the tumor was more prone to be central or peripheral. As there is no consensus in the literature on the quantification of the expression level of GLUT1, MCT1, MCT4 and CAXII with IHC, the "German immunoreactive score" was used [10]. The score was calculated by combining an estimation of the percentage of labeled cells (proportion) with an estimate of the intensity of the labeling (intensity). Regarding the proportion, a lack of labeling was scored at 0, from 1% to 10% of labeled cells was scored at 1, from 11% to 50% at 2, from 51% to 80% at 3 and from 81% to 100% at 4. The intensity was assessed on a scale of 0 to 3, with 0 for negative, 1 for slight, 2 for moderate and 3 for strong staining. Finally, the total score was determined by multiplying the scores of intensity and proportion, the theoretical score ranging from 0 to 12.

As the analysis of CAIX expression by IHC has been the subject of several publications, a method of analysis emerged. The authors defined the cut-off at 85% of labeled cells [11]. For comparison of our results with published data, we used a variant of the "German immunoreactive score" integrating the threshold value (85%) validated by previous studies. The proportion was estimated as 0 in the absence of staining, at 1 for staining of less than 85% and 2 for staining of more than 85%. The intensity was evaluated from 0 to 3 as defined above. The final score was determined by multiplying the scores of intensity and proportion, the theoretical scores ranging from 0 to 6.

### M0 patients for qPCR analysis—Independent qPCR cohort

Tissue samples from 43 patients with non metastatic ccRCC who had undergone surgery in the urology department of the Rennes University Hospital were selected (Table 2). As defined by the 2016 World Health Organization criteria, diagnosis was based upon pathology and cytogenetic analyses.

**Table 2:** Characteristics of the patients included in the survival study–qPCR Cohort.

Variables	qPCR cohort (n = 43)
**Mean age**	63
**Sex**	
**Male**	27 (62.8%)
**Female**	16 (37.2%)
**Furhman grade**	
**II**	20 (46.5%)
**III**	16 (37.2%)
**IV**	7 (16.3%)
**Metastatic status**	
**M0**	43 (100%)
**M1**	0 (0%)

Overall Survival (OS) were calculated from patient subgroups with mRNA levels that were less or greater than the third quartile value. This retrospective study was approved by the institutional review board and was conducted in accordance with the Declaration of Helsinki.

### Gene expression microarray analysis

Normalized RNA sequencing (RNA-Seq) data produced by The Cancer Genome Atlas (TCGA) were downloaded from cbioportal (www.cbioportal.org, TCGA Provisional; RNA-Seq V2). Different parameters were available for 376 non-metastatic ccRCC tumor samples. The results published here are in whole or in part based upon data generated by the TCGA Research Network: http://cancergenome.nih.gov/ [12, 13].

### Statistical analysis

The expression score for each protein in the territory of the highest grade of each tumor was compared to the stage, grade and diameter. The mean and median score of expression of each protein were calculated for each group of Fuhrman II, III and IV. A univariate analysis of the correlation between these parameters was performed using the Kendall nonparametric rank test (inter-tumoral comparison). In some cases, within tumors, a contingent of low-grade (II) was adjacent to a contingent of high-grade (III, IV). In these cases, the expression score for each protein was compared between the two contingents. Comparison of these scores was performed using the Wilcoxon rank-sum test (intra-tumoral comparison). Statistical tests were performed using Statview software. The p was found to be significant below the 0.05 value.

OS was defined as the time from date of diagnosis to the date of death from any cause, censoring those alive at last follow-up. The Kaplan Meier method was used to produce survival curves and analyses of censored data were performed using Cox models.

## Results

### Clinical and histological characteristics

The detailed clinical pathological parameters are reported in Table 1. Median patient age was 63.6 years with 30 males (66%) and 15 females (33%).

### Immunostaining for HIF subunits and HAF

**Fig 1:**
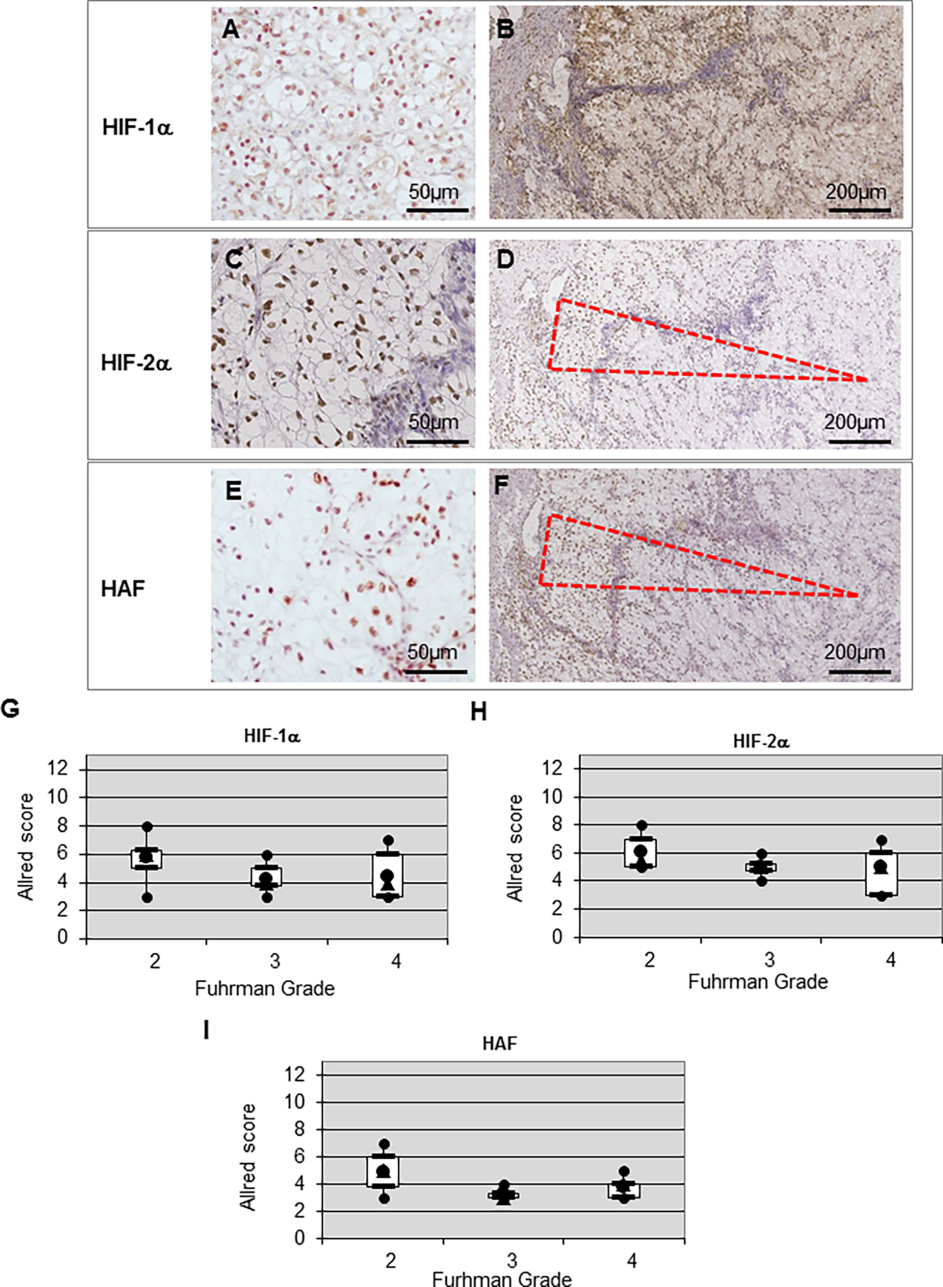
Expression and correlation of HIF-1α, HIF-2α and HAF in ccRCC. (**A, C** and **E**) Positive nuclear staining to HIF-1α, HIF-2α and HAF, respectively, in primary ccRCCs at high magnification (x100). (**B**, **D** and **F**) Heterogenous nuclear staining of HIF-1α, HIF-2α and HAF, respectively, in a tumor at low magnification (x20). (**G**, **H** and **I**) Correlation of HIF-1α, HIF-2α and HAF, respectively, with the Fuhrman grade.

Immunoreactivity to HIF-1α, HIF-2α and HAF was located at the nucleus as shown in the immunostaining depicted in Fig 1A, 1C and 1E, respectively. No specific tissue localization was observed for HIF-1α expression (Fig 1B). Globally, the expression patterns were heterogenous and patchy for HIF-2α and HAF (Fig 1D and 1F). However, expression of HIF-2α was mainly at the invasive front (Fig 1D). Expression of HAF was similar to HIF-2α with higher expression predominating at the periphery of tumor nodules, as represented by the dashed triangle. We confirmed this specific HAF/HIF-2α colocalisation using serial cut sections as observed on Fig 1D and 1F. We observed no exclusivity in HIF-1α (Fig 1B) and HIF-2α (Fig 1D) expression. Moreover, no correlation between HIF-1α, HIF-2α and HAF expression and the Fuhrman tumor grade was found, using the Allred score correlation analysis, as shown on Fig 1G, 1H and 1I.

### Expression status of metabolic actors

**Fig 2:**
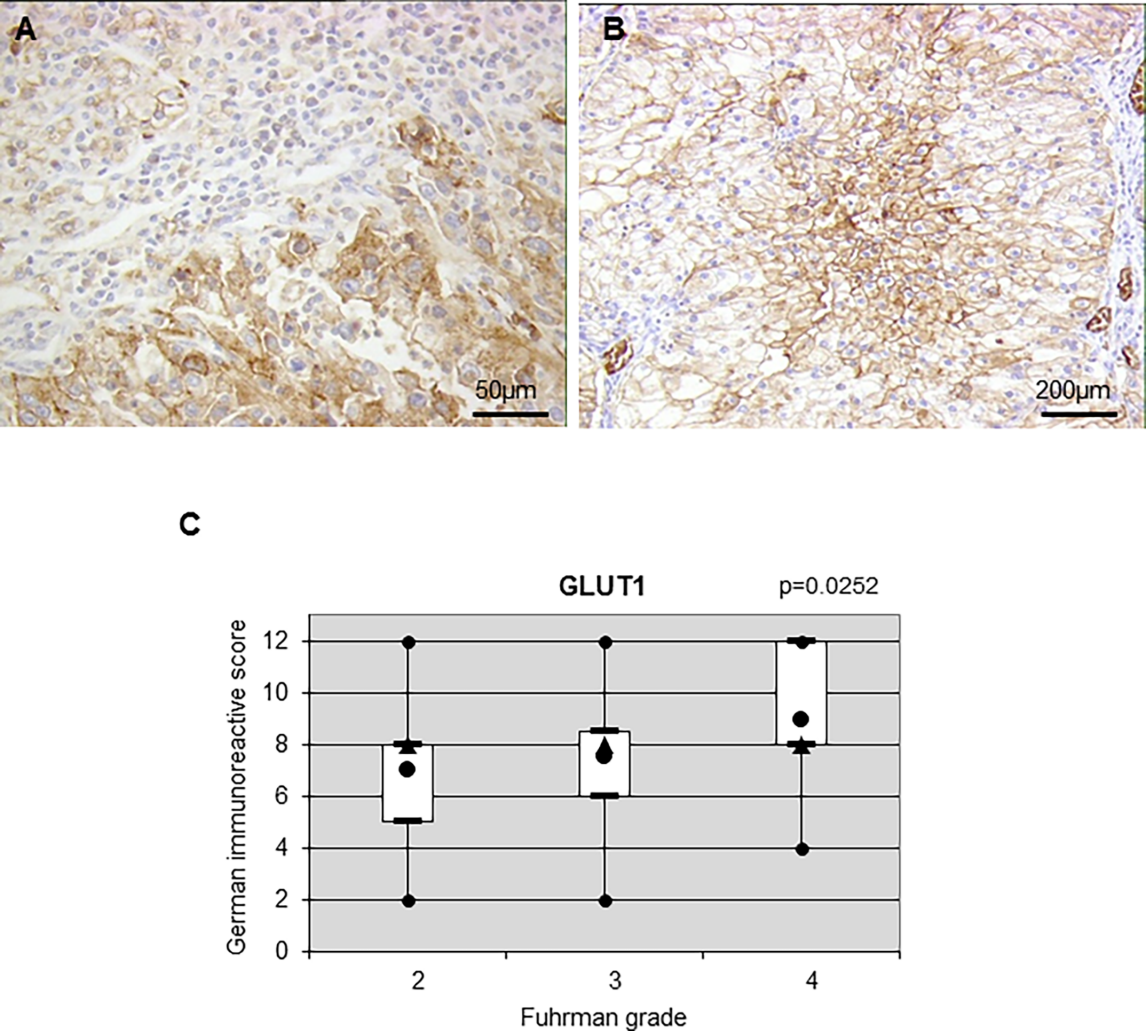
Expression and correlation of GLUT1 in ccRCC. (**A**) Positive membranous staining to GLUT1 in primary ccRCCs at high magnification (x100). (**B**) Heterogenous membranous staining to GLUT1 in a tumor at low magnification (x20). (**C**) Correlation of GLUT1 with the Fuhrman grade.

Expression of the glycolytic marker GLUT1 was mainly located at the cell membrane (membranous) (Fig 2A). Expression was heterogeneous as low (Fig 2B, top left) and high (Fig 2B, bottom right) expression was observed on the same tumor.

Twenty-three patients out of 45 presented contiguous high- and low-grade nodules in the same tumor. Among these patients, 10 presented a higher GLUT1 expression in high-grade nodules and 13 a similar level of expression resulting in significant over expression in high-grade zones (z = 2.803, p = 0.0051) (Table 3). When considering inter-tumor comparison, we showed a statistically significant positive correlation between the Furhman grade and protein expression for GLUT1 (tau = 0.231, p = 0.0252) (Fig 2C and Table 4). We also compared the GLUT1 immunopositivity to the tumor stage (pT) (Table 5) and tumor diameter (Table 6). No significant correlation between these two parameters and expression of GLUT1 was observed suggesting that GLUT1 only positively correlated with the tumor grade.

**Table 3:** Correlation between the expression score of each protein compared to the low-grade contingent (Fuhrman grade II) and the high-grade (Fuhrman grades III and IV) contingent and the characteristics of the immunochemistry using the Wilcoxon rank-sum test.

Parameter	Protein	z	p
Low-grade *versus* high-grade contingents	**GLUT1**	2.803	0.0051
Low-grade *versus* high-grade contingents	**MCT1**	3.180	0.0015
Low-grade *versus* high-grade contingents	**MCT4**	2.521	0.0117
Low-grade *versus* high-grade contingents	**CAIX**	0.255	0.7989
Low-grade *versus* high -grade contingents	**CAXII**	3.059	0.0022

**Table 4:** Correlation between the Fuhrman grade and clinicopathological parameters using the Kendall nonparametric rank test.

Parameter	Protein	tau	p
Fuhrman	**GLUT1**	0.231	0.0252
Fuhrman	**MCT1**	0.448	<0.0001
Fuhrman	**MCT4**	0.137	0.1845
Fuhrman	**CAIX**	-0.104	0.314
Fuhrman	**CAXII**	0.574	<0.0001

**Table 5:** Correlation between pT stage and clinicopathological parameters using the nonparametric rank test of Kendall.

Parameter	Protein	tau	p
pT	**GLUT1**	0.172	0.095
pT	**MCT1**	0.418	<0.0001
pT	**MCT4**	0.254	0.0138
pT	**CAIX**	-0.018	0.8619
pT	**CAXII**	0.342	0.0009

**Table 6:** Correlation between the tumor diameter and the characteristics of the immunochemistry using the Kendall nonparametric rank test.

Parameter	Protein	tau	p
Diameter	**GLUT1**	0.149	0.1489
Diameter	**MCT1**	0.274	0.008
Diameter	**MCT4**	-0.093	0.3701
Diameter	**CAIX**	0.188	0.0687
Diameter	**CAXII**	0.32	0.0019

**Fig 3:**
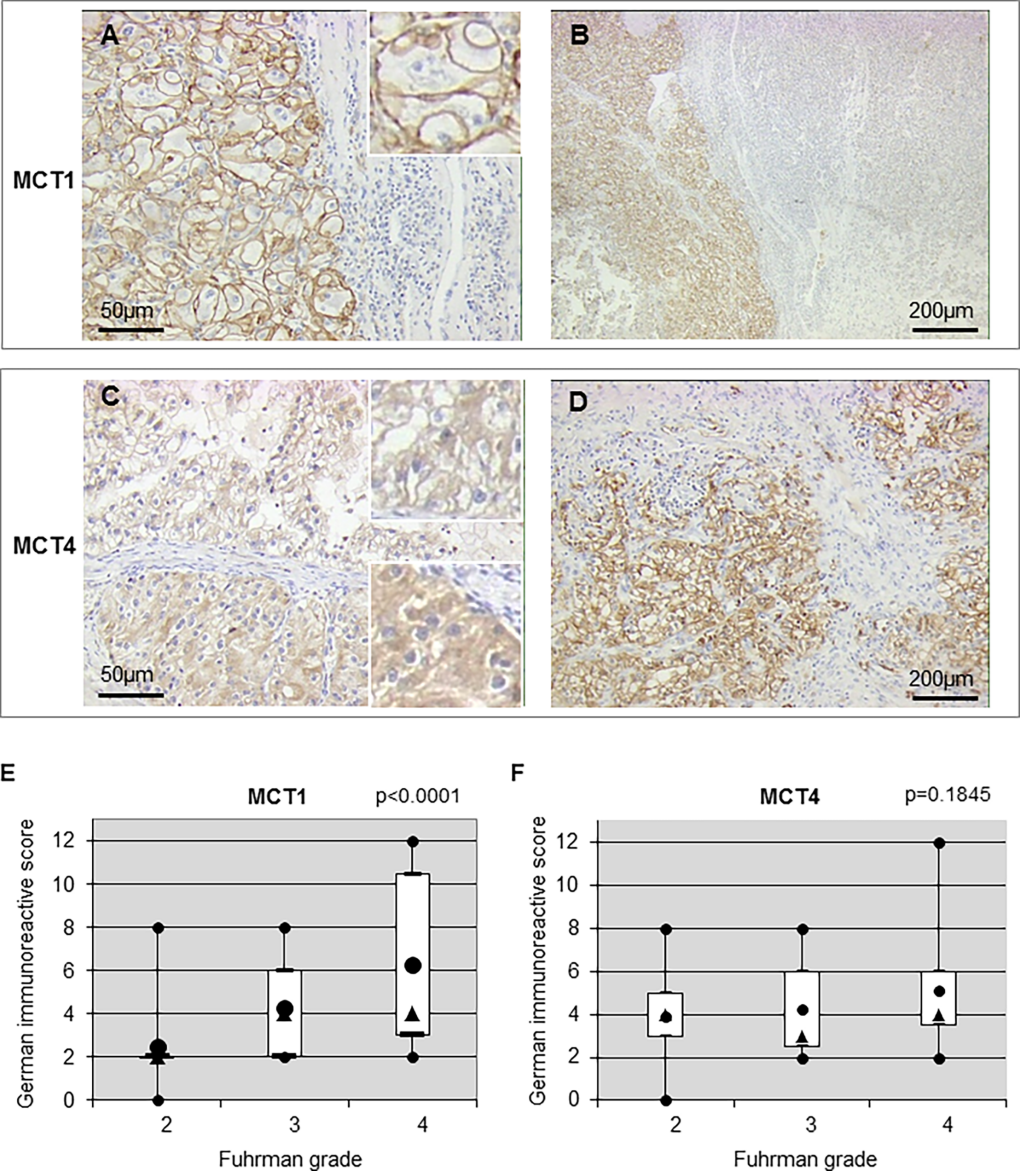
Expression and correlation of MCT1 and MCT4 in ccRCC. (**A** and **C**) Positive membranous staining to MCT1 and MCT4 in primary ccRCCs at high magnification (x100). (**B** and **D**) Heterogenous membranous staining to MCT1 and MCT4 in a tumor at low magnification (x20). (**E** and **F**) Correlation of MCT1 and MCT4, respectively, with the Fuhrman grade.

MCT1 (Fig 3A) and MCT4 (Fig 3C) showed a clear membranous staining pattern with substantial variation in intensity and extent (Fig 3B and 3D). However, cytoplasmic staining for MCT4 also appeared in high-grade tumors (Fig 3C—bottom right insert) in the same tumor expressing membranous staining (Fig 3C—top right insert).

Similarly to GLUT1, among the 23 tumors with contiguous high- and low-grade nodules, 13 and 8 presented, respectively a significantly higher expression in high-grade zones for MCT1 (z = 3.180, p = 0.0015) and MCT4 (z = 2.521, p = 0.0117) (Table 3). The strongest correlation was observed for MCT1 with the Fuhrman grade (tau = 0.448, p< 0.0001) (Table 4 and Fig 3E) whereas no correlation was found between MCT4 and the aggressive phenotype (Table 4 and Fig 3F). Moreover, positive correlations between tumor stage and MCT1 (tau = 0.418, p≤0.0001) or tumor stage and MCT4 (tau = 0.254, p = 0.0138) were obtained (Table 5). MCT1 also correlated to diameter (tau = 0.274, p = 0.008) (Table 6). Taken together, these results strongly suggest an important role for MCT1 in ccRCC tumor aggressiveness compared to MCT4.

**Fig 4:**
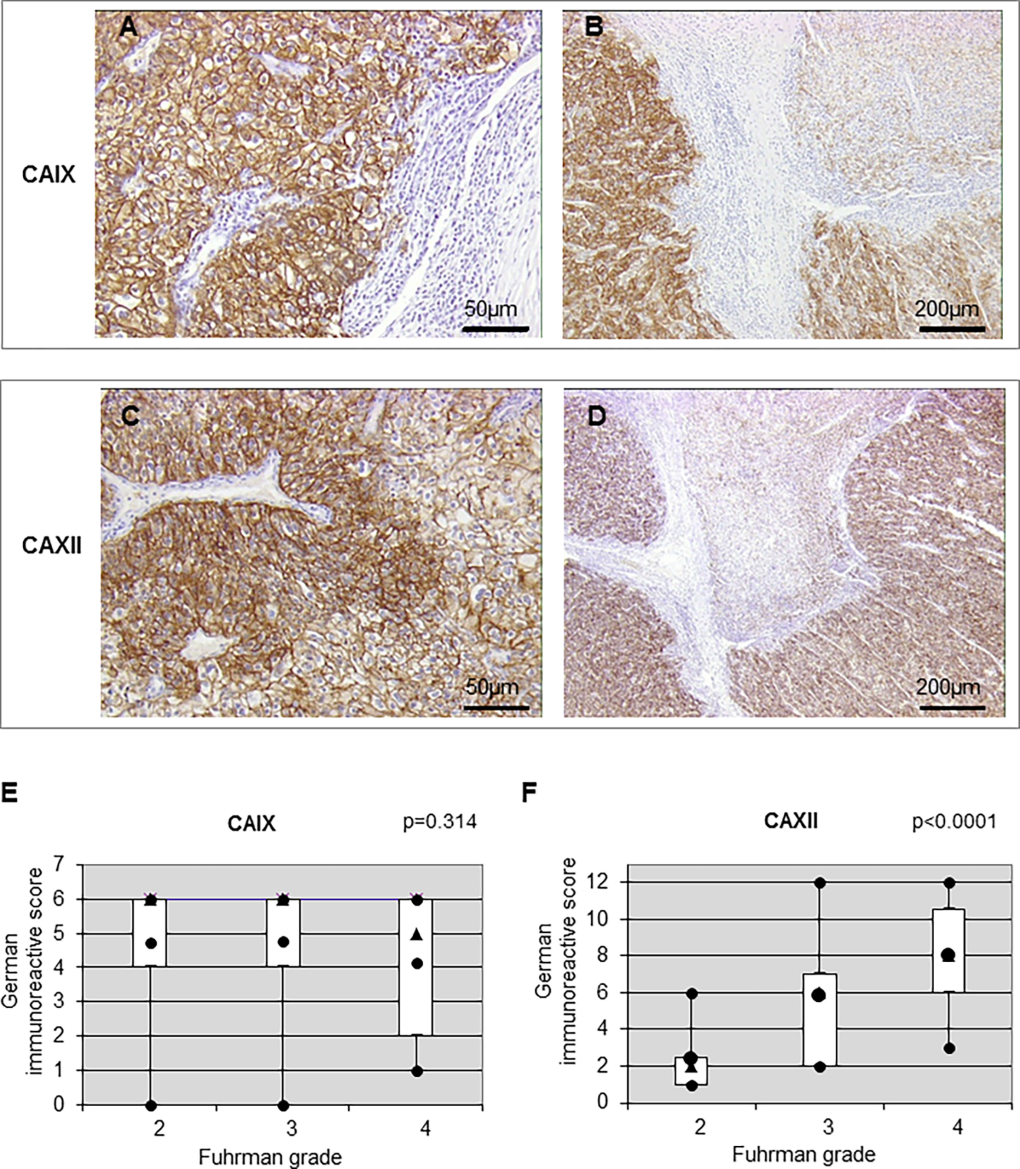
Expression and correlation of CAIX and CAXII in ccRCC. (**A** and **C**) Positive membranous staining to CAIX and CAXII in primary ccRCCs at high magnification (x100). (**B** and **D**) Heterogenous membranous staining to CAIX and CAXII in a tumor at low magnification (x20). (**E** and **F**) Correlation of CAIX and CAXII, respectively, with the Fuhrman grade.

Expression of CAIX and CAXII was located at the plasma membrane (Fig 4A and 4C) and presented variation in intensity (Fig 4B and 4D).

We found high- and low-expressing areas in the same tumor. Among the 23 tumors with contiguous high- and low-grade nodules, 12 presented significantly higher CAXII expression (z = 3.059, p = 0.0022) (Table 3). For intra-tumor comparison, no correlation was found when comparing the low-grade and high-grade contingent (Table 3). Fig 4E and 4F represent association between CA expression and tumor grade. The association between CAIX and the Furhman grade did not reach statistical significance (tau = -0.104, p = 0.314) (Fig 4E and Table 4). However, a statistically significant correlation between the CAXII level of expression and the Fuhrman grade was found (tau = 0.574, p<0.0001) (Fig 4F and Table 4). Moreover, the MCT1 and CAXII levels of expression presented a positive correlation with pT (tau = 0.342, p = 0.0009) (Table 5) and diameter (tau = 0.32, p = 0.0019) (Table 6), whereas no correlation was found for CAIX (Tables 5 and 6).

Taken together, these results highlight for the first time three new markers linked to the Fuhrman grade and thus to the aggressiveness of the ccRCC: including GLUT1, MCT1 and CAXII.

### Overexpression of GLUT1 and MCT1 correlated with reduced overall survival of non-metastatic RCC patients

Analysis of online available data (TCGA) showed co-occurrence of MCT1 and GLUT1 (p<0.001) and MCT1 and CAXII (p = 0.003) mRNA expression in samples from non-metastatic RCC patients (S1 Table). Analysis of the same cohort showed that GLUT1 (p<0.001), MCT1 (p<0.01) and CAXII (p<0.001) mRNA expression correlated with the Fuhrman grade, while MCT4 and CAIX mRNA expression did not correlate with the Fuhrman grade (S1 Fig). This analysis corroborates our results as demonstrate before for the protein level.

Next, we analyzed the impact of the mRNA expression on OS in the mRNA cohort (Table 2) and in the TCGA mRNA cohort.

**Fig 5:**
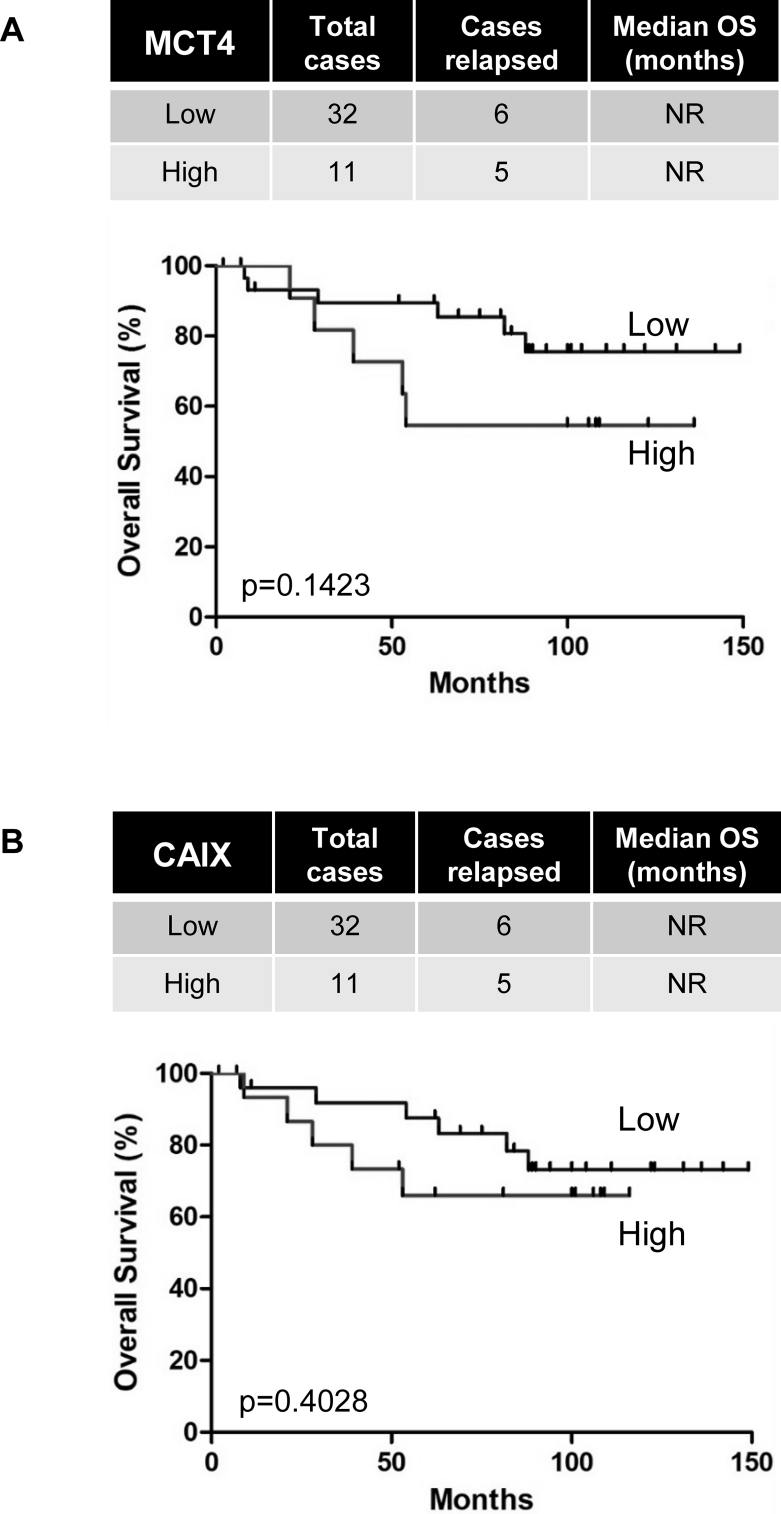
Overexpression of MCT4 and CAIX did not correlate with reduced overall survival of non-metastatic ccRCC patients. Kaplan–Meier analysis of OS of M0 patients. OS was calculated from patient subgroups with mRNA levels that were less or greater than the third quartile. Statistical significance (*p* values) is indicated.

MCT4 and CAIX mRNA and protein expression did not correlate with the Fuhrman grade as described before. As expected, we found that MCT4 and CAIX mRNA levels did not correlate with OS in both cohorts (Fig 5 and S2 Fig).

**Fig 6:**
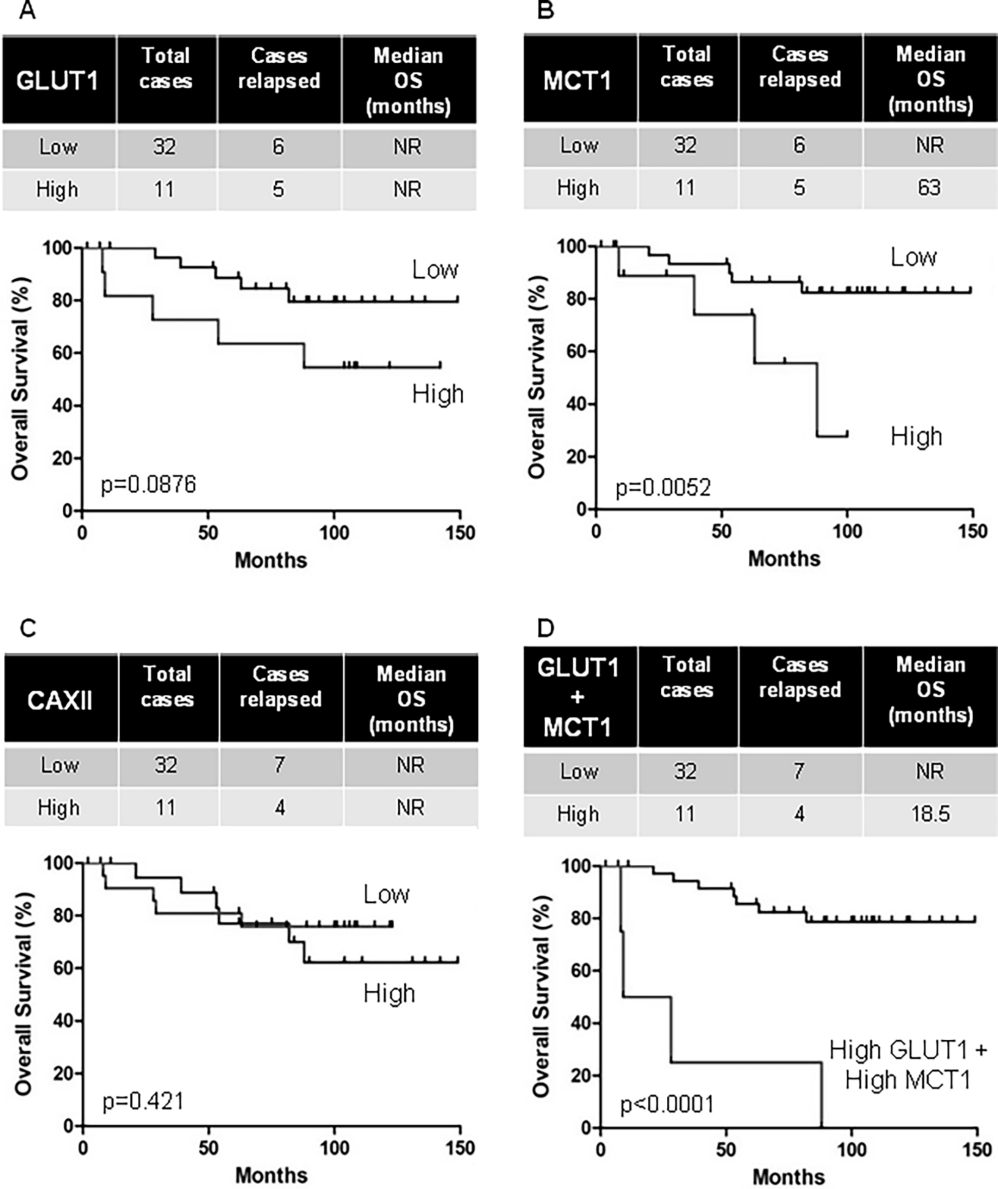
Overexpression of GLUT1 and MCT1, but not CAXII, correlated with reduced overall survival of non-metastatic RCC patients. Kaplan–Meier analysis of OS of M0 patients. OS was calculated from patient subgroups with mRNA levels that were less or greater than the third quartile. Statistical significance (*p* values) is indicated.

GLUT1, MCT1 and CAXII mRNA and protein expression correlated with the Fuhrman grade as described before. As expected, overexpression of GLUT1 and MCT1 mRNA correlated with reduced OS. Surprisingly, this was not the case for CAXII (Fig 6). MCT1 mRNA (median survival: 63 months vs not reached, p = 0.0052) in the tumors of M0 patients correlated with shorter OS. We observed the same trend with GLUT1 mRNA (median survival: not reached, p = 0.0876). The same results were found with the TCGA cohort, high GLUT1 mRNA (median survival: 79.5 months vs not reached, p = 0.004) and high MCT1 mRNA (median survival: 79.5 months vs not reached, p = 0.007) correlated with shorter OS (S3 Fig). Moreover, discrimination of patients with a concomitant high level of GLUT1 mRNA and MCT1 mRNA had a substantially shorter OS (median survival: 18.5 months vs not reached, p = 0.0001, Fig 6).

## Discussion

Understanding the heterogeneity of ccRCC and the respective role of the different molecular phenotypes is fundamental to determining the precise prognosis and the tumor sensitivity to drugs in addition to defining potential therapeutic targets. While the impact of the Fuhrman grade on prognosis is well established and widely integrated into clinical practice it is underpinned by unknown mechanisms.

To answer this problematic, we first provided topographic data from the analysis of entire slides. This analysis is original and superior to analysis on tissue microarrays for several reasons. On the one hand, it allows evaluation of the protein expression on a large number of cells, which increases its reliability. On the other hand, intra-tumor heterogeneity is thus visible on different contingents of the same tumor, which have different characteristics and grades. For example, GLUT1 overexpression has been correlated to poor prognosis [14]. However, data in the literature concerning correlation between the GLUT1 expression level and tumor grade are contradictory [15, 16]. These dissimilarities are certainly due to methodological discrepancies. Our studies into whole sections clearly demonstrated the heterogeneity of GLUT1 expression. Moreover, high expression of GLUT1 correlated with poor OS in our small cohort (43 patients). Although this result was not statistically significant, it was reinforced by the TCGA cohort (376 patients) by showing a statistically significant correlation. Overexpression of CAIX is also common in solid cancers and is a poor prognostic factor, except in ccRCC. However, studies carried out so far reported discordant results [17, 18]. In our study, we did not observe correlation between CAIX expression (protein and mRNA) and tumor grade or OS. We only observed a marked heterogeneity for CAIX expression, depending on the tumor area, which questions the relevance of previous studies based on tissue microarrays. Although CAXII overexpression has been observed in many cancers and in ccRCC, its prognostic value remains questionable. Our study clearly shows that CAXII is a central biomarker, as we observed a strong correlation between the Fuhrman grade and CAXII expression (protein and mRNA). However, differences between CAIX and CAXII expression remain unexplained, which may reveal a dual role for these two direct HIF targets. Furthermore, CAXII expression did not correlate with survival or prognosis. CAXII appears to be a simple marker of aggressiveness (tumor grade). Finally, both MCT1 and MCT4 are also highly expressed in tumors comparatively to matched normal tissue and often correlate with poor prognosis [19, 20]. Moreover, clinical evidence demonstrated that the lactate produced by tumors correlated with poor prognosis and resistance to radiotherapy [21]. Kim *et al*. provided a simultaneous analysis of MCT1 and MCT4 in ccRCC and demonstrated that overexpression predicted progression free survival [22]. Our study confirmed these results for MCT1 and for the first time directly correlated MCT1 with tumor grade, consistent with that reported previously for cervix carcinoma [23]. This central role of MCT1 also concerns prognosis, the mRNA level of MCT1 correlated with OS. However, high mRNA levels of GLUT1 and MCT1 had a strong prognostic value. Indeed, the median survival of M0 patients with a tumor strongly expressing these two markers was about 18.5 months whereas in the rest of the population it was greater than 150 months.

In our global analysis, we observed heterogeneous expression of GLUT1, MCT1, MCT4, CAIX and CAXII, key molecular markers of the pH machinery, within tumor and between tumors. Several scenarios could be proposed to explain these differences: (i) As ccRCC is defined by the inactivation of the *vhl* gene and thus by stabilization of HIF-α isoforms, different gene profils could be observed depending on which HIF-α isoform is stabilized. Raval *et al*. have shown two expression profiles of HIF-α isoforms *in vitro* in ccRCC depending on either HIF-1 and HIF-2 or HIF-2 alone [24]. Klatte *et al*. found no correlation between HIF-1α expression and the Fuhrman grade [25], as observed in our study with both HIF-1α and HIF-2α. The patchy localization of HIF-1α, the isoform involved in the control of genes involved in metabolism, may partly explain the heterogeneity of expression of the markers we studied. (ii) Furthermore, the heterogeneous pattern of protein expression found in different tumor areas suggested that the protein expression could be modulated by variable local vascularization or conditions of pH inside the tumor. Such modes of regulation have already been discussed for CAIX [26, 27]. However, this latter scenario probably does not explain our results for GLUT-1. (iii) The appearance of lobulation observed for some markers suggests the existence of different cell populations. It has been shown that higher-grade lesions were characterized by superadded genetic abnormalities [28]. It will be interesting to study more specifically the impact of oncogenes on GLUT-1, CAXII and MCT1 expression.

Interestingly, high expression of HIF-2α and HAF has been observed at the invasive front. This overexpression is similar to that of already reported overexpression for MMP-1, a HIF-2-induced protein, in the periphery of ccRCC [29], which contributes to degradation of the extracellular matrix to facilitate invasion [30]. Our observations could provide a better understanding of tumor invasion and highlights HAF and HIF-2α as potential markers to define histological assessment of the margin, especially for nephron sparing surgery. The preferential expression of HIF-2α in cells localized at the invasive front is consistent with the differential and non-redundant roles of the two isoforms of HIFα.

Moreover, our observations concerning HAF are consistent with the results of Koh *et al*. [31]. To our knowledge, our study is the first to evaluate the simultaneous expression of HIF-1α, HIF-2α and HAF in tumors from the same patients with ccRCC.

Interplay between glycolysis and oxidative phosphorylation (OXPHOS) permits tumors to adapt their production of energy to the microenvironmental changes and energetic requirements of the tumor. Despite the observed decrease in the mitochondrial content of tumors, cancer cells maintain a significant level of OXPHOS to rapidly switch from glycolysis to OXPHOS during carcinogenesis [32]. Moreover, ccRCC have already been described to perform functional glycolysis and OXPHOS [33].

HIF-1α is responsible for the regulation of genes encoding enzymes involved in the glycolytic pathway but HIF-2α target genes are involved in invasion, for example the matrix metalloproteinases. In our study, the expression of HIF-1α within the tumor correlated with the high level of glycolysis previously described in ccRCC. However, at the invasive front of the tumor HAF was co-expressed with HIF-2α but not with HIF-1α. In these cells, and according to the literature, we can thus hypothesize that HAF decreased the HIF-1α level inducing a decrease in glycolysis and in the meantime a stabilization of HIF-2α. Moreover, HIF-2α (but not HIF-1α) has been shown to cooperate with a number of oncoproteins frequently deregulated in cancer, such as c-Myc, epidermal growth factor receptor, and K-Ras and promoted tumor aggressiveness, EMT and invasion [4, 24]. Finally, HAF and/or HIF-2 can increase OXPHOS (*via* c-Myc and K-Ras, [32]) and higher reactive oxygen species production to induce invasion and metastasis [34]. Recently, it has been shown that, in terms of metabolism, there is an inverse correlation between adenosine monophosphate-activated protein kinase (AMPK) (linked to the OXPHOS state) and HIF-1α (linked to the glycolytic state) [35]. Our study questions the role of metabolism in tumor development of RCC. It would be of interest, for example, to examine the link between HAF / HIF-2α and AMPK / OXPHOS of cells at the invasive front.

A correlation between cell proliferation and tumor grade has already been reported in ccRCC [36, 37]. As cells proliferate, their needs in energy increases justifying an increase in expression of proteins involved in glycolysis, such as GLUT1. Therefore, as glycolysis increases, lactate production and intracellular acidification are enhanced increasing expression of proteins involved in pH regulation, such as MCT1, MCT4, CAIX and CAXII. Indeed, inter-tumor comparisons showed a significant positive correlation between the level of mRNA and protein expression of MCT1, GLUT1, CAXII and the Fuhrman grade. These results are especially robust as they are demonstrated in two cohorts with two different and complementary techniques. We also observed a significant positive correlation between the protein expression levels of MCT1, MCT4, CAXII and pT, and the level of MCT1 and CAXII expression and diameter of the tumor. Moreover, intra-tumor comparison revealed significantly higher expression of MCT1, MCT4, GLUT1 and CAXII in the higher grade contingent.

Our results clearly show for the first time that the most powerful histoprognostic parameter, the Fuhrman grade, can be partly connected to metabolic markers and more precisely to strong changes in metabolism. These results validate our hypothesis that tumor aggressiveness is related to the metabolic switch and therefore to a high level of glycolysis. Therefore, we propose that association of GLUT1/MCT1 with the Fuhrman grade could be used as a potent tool to precisely characterize ccRCC patients.

## Supporting information

S1 Table Co-occurrence of MCT1, GLUT1 and CAXII mRNA expression in non-metastatic RCC patients. Analysis of cbioportal databases highlighted the levels of mRNA in non-metastatic RCC. These results are in whole or in part based upon data generated by the TCGA Research Network. Statistical significance (*p* values) are indicated.
(TIFF)

S1 Fig Overexpression of GLUT1, MCT1 and CAXII correlated with Fuhrman grade of non-metastatic RCC patients. Analysis of cbioportal databases highlighted the levels of mRNA in non-metastatic RCC, and Furhman grade. These results are in whole or in part based upon data generated by the TCGA Research Network. Statistical significance (*p* values) are indicated.
(TIFF)

S2 Fig Overexpression of MCT4 and CAIX not correlated with reduced overall survival of non-metastatic RCC patients. Kaplan-Meier survival curves of non-metastatic (M0) RCC patients with high or low mRNA expression. These results are in whole or in part based upon data generated by the TCGA Research Network. Statistical significance (*p* values) are indicated.
(TIFF)

S3 Fig Overexpression of GLUT1 and MCT1, but not CAXII, correlated with reduced overall survival of non-metastatic RCC patients. Kaplan-Meier survival curves of non-metastatic (M0) RCC patients with high or low mRNA expression. These results are in whole or in part based upon data generated by the TCGA Research Network. Statistical significance (*p* values) are indicated.
(TIFF)
